# Effects of Lactic Acid Bacteria Isolated From Rumen Fluid and Feces of Dairy Cows on Fermentation Quality, Microbial Community, and *in vitro* Digestibility of Alfalfa Silage

**DOI:** 10.3389/fmicb.2019.02998

**Published:** 2020-01-09

**Authors:** Linna Guo, Dandan Yao, Dongxia Li, Yanli Lin, Smerjai Bureenok, Kuikui Ni, Fuyu Yang

**Affiliations:** ^1^College of Grassland Science and Technology, China Agricultural University, Beijing, China; ^2^College of Animal Science and Technology, Yangzhou University, Yangzhou, China; ^3^Beijing Sure Academy of Biosciences, Beijing, China; ^4^Department of Agricultural Technology and Environment, Rajamangala University of Technology Isan, Nakhon Ratchasima, Thailand

**Keywords:** lactic acid bacteria, rumen fluid, feces, alfalfa silage, microbial community, *in vitro* digestibility

## Abstract

The objective of this study was to select lactic acid bacteria (LAB) isolated from the rumen fluid and feces of dairy cows, and evaluate their effects on silage quality of alfalfa after 30 or 60 days of ensiling. One hundred and four LAB strains were isolated from rumen fluid and feces of six dairy cows, of which four strains (*Lactobacillus plantarum* F1, *L. plantarum* F50, *Lactobacillus salivarius* L100, and *Lactobacillus fermentum* L120) and one commercial inoculant (GFG) isolated from forage were employed for further study. The silages treated with F1 had the lowest (*P* < 0.05) pH value and the highest (*P* < 0.05) lactic acid (LA) content in all treatments. Besides, higher (*P* < 0.05) *in vitro* digestibility was also observed in F1-treated silage after 60 days of ensiling. The microbial analysis showed that the *Lactobacillus* abundance in the F1-treated silages increased to 60.32%, higher than other treatments (5.12–47.64%). Our research indicated that strain F1 could be an alternative silage inoculant, and dairy cows could be a source for obtaining excellent LAB for ensiling.

## Introduction

Ensiling as a traditional method for preserving fresh forage provides animals with high nutritious feeds throughout the year (Burns et al., 2018). During the fermentation process, lactic acid bacteria (LAB) multiply under anaerobic condition, and could ferment WSC into lactic acid (LA) and other acids (Yang et al., 2019). To obtain high-quality feeds for livestock, LAB are often employed as silage inoculants.

Currently, most of LAB inoculants for ensiling were isolated from plant-derived materials, including fresh forage, silage, and so on (Zhang et al., 2015, 2018; Ni et al., 2017b; Wang et al., 2017). However, plant-derived LAB inoculants sometimes cannot improve animal performance (Kristensen et al., 2010; Santos et al., 2017; Silva et al., 2017). For example, Khota et al. (2017) reported that although LAB from sweet corn stover silage could improve the silage quality, it did not show positive effect on *in vitro* dry matter (DM) digestibility and gas production (GP) of beef cattle.

Moreover, Timmerman et al. (2006) showed that LAB isolated from the gut of host animal could be more beneficial on animal performance than LAB from other sources. As a major component of the gastrointestinal microflora, diverse LAB exist in the digestive tract of animals. Vlková et al. (2006) isolated various LAB strains from calf rumen, such as *Bifidobacterium*, *Lactobacillus*, and *Enterococcus*. Krause et al. (2003), Hernandez et al. (2008), and Nader-Macías et al. (2008) detected *Lactobacillus vitulinus*, *Lactobacillus johnsonii*, *Lactobacillus murinus*, *Lactobacillus ruminis*, and *Streptococcus bovis* in animal guts. Furthermore, *Lactobacillus plantarum* and *Pediococcus acidilactici* isolated from calf feces could be effective in promoting animal performance (Timmerman et al., 2005; Rodriguez-Palacios et al., 2009).

Animal-derived LAB may provide a new approach for the development of silage inoculant. As we know, LAB inoculants usually dominate silage fermentation, therefore silage could be used as a vehicle to convey inoculated LAB (Han et al., 2014). We hypothesized that LAB from animals not only could improve the silage quality, but also be more likely to have a good environmental adaptability in animals, subsequently improving animal performance. Previous research showed that *Enterococcus faecium* isolated from yak rumen could be exploited for reducing the lignocellulosic biomass of grass silage (Li et al., 2018c). However, until now, little information is available about the effects of LAB derived from dairy cow on silage quality. In this study, we selected LAB strains from rumen fluid and feces of dairy cow fed with alfalfa silage for almost 1 year. Then, we evaluated their effects on fermentation quality, microbial community, and *in vitro* digestibility of alfalfa silage. The results of this study were expected to enrich the resource of LAB inoculants for ensiling.

## Materials and Methods

### Isolation, Screening, Characterization, and Identification of LAB Isolated From Rumen Fluid and Feces of Dairy Cows

The rumen fluid and feces samples were collected from 6 (100–200 d) Holstein dairy cows in good condition and restricted to the supply of the same amount of alfalfa silage in Beijing Breeding Stock of Dairy Cattle Science and Technology Park (Shunyi District, Beijing, China). The animal care and use was conducted in accordance with practices outlined in the Guide for the Care and Use of Agriculture Animals in Agriculture Research and Teaching (FASS, 2010).

Each sample volume of rumen fluid was 100 mL and the weight of feces was 100 g. To remove solid phase, 1 mL rumen fluid flited with four layers of gauze was inoculated on 10 mL MRS (de Man, Rogosa, Sharpe, Beijing Aoboxing Bio-tech Co., Ltd., Beijing, China) liquid medium at 30°C. 1% (V/V) inoculum was sub-cultured once every 24 h, and the 0.1 mL culture of fifth generation was treated with a 10-fold gradient dilution. The 0.5 g feces were blended with 4.5 mL of sterilized water, and then serially diluted to 10^–6^. Then dilutions of rumen fluid and feces were plated on MRS agar medium at 30°C for 48 h under anaerobic conditions to isolate and purify LAB, respectively. All isolated strains were cultivated on MRS agar medium and their morphological characteristic, growth capacity, and acid-producing ability were determined. The LAB strains with fast growth capacity and high acid-producing ability were tested for their physiological and biochemical characteristics. And 16S rRNA gene sequencing was employed to identify the screened strains genetically (Zhang et al., 2014).

### Silage Making

Alfalfa (*Medicago sativa*, WL363HQ) forages were harvested in early bloom stage of second cutting in the experimental plot of the Teaching Experiment Field of China Agricultural University, Zhuozhou, China (39°28′N, 115°510′E). The plant materials were wilted to about 60% moisture and chopped into segments at a theoretical length of 20 mm. Silages were prepared using a small-scale system: approximately 200 g portions of chopped alfalfa were packed into plastic film bags (Hiryu KN type; 18 × 26 cm; Asahi Kasei, Tokyo, Japan). The ensiling materials were treated with no additive as control, with a commercial inoculant (GFG, *L. plantarum* isolated from forage, Sichuan Gao Fuji Biotechnology Co., Ltd., Sichuan, China) or with one of the four selected strains (*L. plantarum* F1, *L. plantarum* F50, *Lactobacillus salivarius* L100, and *Lactobacillus fermentum* L120) at 1.0 × 10^6^ colony forming units (cfu)/g of fresh matter (FM), respectively. The bags per treatment were performed in triplicate. Then bags were vacuum-sealed and stored at room temperature around 25°C.

### Analysis of Microbial Population, Fermentation Quality, and Chemical Composition

The pre-ensiled materials and silage samples after closure of the plastic pouches were collected for analysis. Sub-samples (10 g) were blended with 90 ml of sterilized water and serially diluted from 10^–1^ to 10^–5^. Then, 20 μl of dilution at 10^–1^, 10^–3^, and 10^–5^ were spread onto the corresponding agar separately. The number of LAB was counted on MRS agar mediums and kept in anaerobic incubator at 30°C for 48 h, and molds and yeasts were incubated in general incubator on Rose Bengal Agar mediums at 28°C for 48 h (all mediums were obtained from Beijing Aoboxing Bio-tech Co., Ltd., Beijing, China). Colonies were measured by plate count as viable numbers of microorganisms in cfu/g of FM.

A 10^–1^ dilution of sample was filtered with 0.22 μm filter paper, and the pH value was measured by pH meter (PHS-3C, INESA Scientific Instrument, Shanghai, China). The filtrate was used to determine the ammonia–nitrogen (NH_3_–N), LA, acetic acid (AA), propionic acid (PA), and butyric acid (BA). The NH_3_–N content was determined by phenol–hypochloric acid colorimetry provided by Broderick and Kang (1980). Fermentation quality including LA, AA, PA, and BA contents were determined by high-performance liquid chromatography (HPLC) (LC-20A; Shimadzu, Tokyo, Japan) (Tian et al., 2017).

The pre-ensiled materials and silage samples were dried at 65°C for 48 h to test DM content by oven, and milled to pass through a 1.0 mm screen for determination of chemical composition and *in vitro* rumen fermentation. Total nitrogen content (N) was determined by the Kjeldahl procedure and CP was calculated by multiplying N with 6.25 (AOAC, 1990). Neutral detergent fiber (NDF) and acid detergent fiber (ADF) contents were determined by the method of Van Soest et al. (1991). Water soluble carbohydrates (WSC) contents were determined using the anthrone method (Tian et al., 2017).

### DNA Extraction, 16S rRNA Gene Amplification, and High-Throughput Sequencing

The pre-ensiled alfalfa and silage samples were added into the sterile 0.9% NaCl solution, and then treated with table concentrator at 120 r/m for 2 h. Filtered through two layers of medical gauze. The filtrates were centrifuged at 12,000 r/m for 15 min at 4°C. The total DNA was extracted from resulting cell pellets (Ni et al., 2017a).

The content of DNA was amplified by the bacterial 16S rRNA primers targeting the V3–V4 regions of 338F (ACTCCTAC GGGAGGCAGCAG) and 806R (GGACTACHVGGGTWT CTAAT). PCR condition was set as follows: initial denaturation at 95°C for 2 min, 25 cycles of denaturation at 95°C for 30 s, annealing at 55°C for 30 s, elongation at 72°C for 30 s, and extension at 72°C for 5 min. Each treatment was conducted in triplicate and the mixture was purified to perform sequencing (Zheng et al., 2017). Sequencing of DNA samples was conducted with Majorbio Bio-Pharm Technology Co., Ltd. (Shanghai, China) on an Illumina MiSeq 2 × 250 platform. Any sequences which contained mismatches and ambiguous reads in the primers were removed for quality-control purposes. Chimeric sequences were identified and discarded using UCHIME, and the obtained high quality reads were clustered into individual OTUs using UPARSE^1^ (version 7.1) at a default similarity level of 97% (Amato et al., 2013). Shannon diversity index, Chao richness estimator, Simpson evenness index, and Good’s coverage were computed using Mothur^2^ (version 1.30.1) for analysis of alpha-diversities. OTUs were classified using Ribosome Database Project (RDP) Classifier^3^ against the SILVA (SSU115)16S rRNA database with a minimum confidence cut-off of 0.7 and then denominated at the genus levels. Principal component analysis (PCA) was performed using the PLS Toolbox software package v.6.2.1, for MATLAB 7.12.0. Prior to the redundancy analysis (RDA) was analyzed using detrended correspondence analyses that conducted by the R software package^4^ (ver. 3.2.5) (Tian et al., 2017).

### *In vitro* Digestibility of the Silages Ensiled for 60 Days

The rumen fluid samples were collected from three fistulated Holstein dairy cows 1 h before the morning feeding. After the collection, the rumen fluid was collected and filtered with four layers of gauze. The temperature of the sample was maintained at 39°C in water bath, and CO_2_ was continuously added to maintain the anaerobic environment. Briefly, 0.2000 g (accurate to 0.0001 g) dried silage powder sample was loaded into a fermentation bottle (volume 120 mL); each sample was set five parallels, and another three blanks, and the whole experiment was repeated in three runs. Twenty-five milliliter of rumen fluid and 50 mL of pre-warmed buffer (pH 6.80) were well mixed (Li D.X. et al., 2018). N_2_ was passed for 5 s to eliminate the bottle air, and the bottle was quickly sealed with rubber stopper and knob. All bottles were cultured continuously at 39°C for 72 h to record GP (GP72) by AGRS-III Microorganism Fermentation Micro Recorder. The fermentation broth was collected after filtration with a nylon bag (8 × 12 cm, 42 μm, 300 mesh). The nylon bag was rinsed with tap water until the water was clear, then washed twice with distilled water, and dried to constant weight at 65°C for *in vitro* DM disappearance (IVDMD) (Wang et al., 2019). Formula (1) was used for calculation of cumulative GP (*t*, mL/g DM), where *A* is the asymptotic GP generated at a constant fractional rate (*c*) per unit time, *t* represents the gas recording time (h), and Lag is the lag time phase (h) prior to the initiation of GP (France et al., 2000). Formula (2) was used for calculation of average GP rate (AGPR, mL/h).

(1) $${\text{GP}}_{t}=A^{\prime }\,\left[1-e^{-c^{\prime }\,\left(t-\text{Lag}\right)}\right]$$

(2) $$\text{AGPR}=\frac{A\times c}{2\times \left(\text{Ln}\,2+\text{c}\times \text{Lag}\right)}$$

### Statistical Analysis

All the statistical analyses were performed using SAS 9.0 software (SAS Institute, Cary, NC, United States, 2002) and the results represented by their mean values. Duncan’s multiple range method was used to determine the significant difference between means. The level of statistical significance was declared at *P* < 0.05 and *P* < 0.01.

## Results

### Characterization and Identification of Selected LAB Strains

A total of 104 LAB strains were isolated from rumen fluid and feces. The characterizations of four selected strains in this study were shown in Table 1. F1 and F50 were isolated from feces, while L100 and L120 were isolated from rumen fluid of dairy cows. F1, F50, and L100 were gram-positive, catalase-negative, and rod homo-fermentative, and L120 was gram-positive and catalase-negative rod hetero-fermentative. L120 could grow at the temperature from 15 to 40°C, while other three strains could grow at 5–45°C. F1, F50, and L100 could grow at pH ranged from 3.0 to 9.0 and below NaCl concentration of 3.0% (w/v), only F1 could grow at NaCl concentration of 6.5% (w/v). All of the strains could produce acid from cellobiose, maltose, saccharose, and D-raffinose. F1 and F50 could produce acid from esculine and sorbitol. However, except L120, other strains could produce acid from mannitol and salicin. F1 and F50 were placed in the *L. plantarum* cluster, L100 was placed in *L. salivarius* cluster, and L120 was placed in the *L. fermentum* cluster with 16S rRNA sequence similarities >99%.

**TABLE 1:** Morphological, physiological, and biochemical properties of selected lactic acid bacteria.

**Items**	**Strains**			
	**F1**	**F50**	**L100**	**L120**
Genus	*Lactobacillus plantarum*	*Lactobacillus plantarum*	*Lactobacillus salivarius*	*Lactobacillus fermentum*
Shape	Rod	Rod	Rod	Rod
Fermentation type	Ho	Ho	Ho	He
Gram strain	+	+	+	+
Catalase activity	−	−	−	−
Gas from glucose	−	−	−	+
**Growth at temperature (°C)**				
5	+	+	+	−
10	+	+	+	−
15	+	+	+	+
20	+	+	+	+
25	+	+	+	+
30	+	+	+	+
35	+	+	+	+
40	+	+	+	+
45	+	+	+	−
50	+	−	−	−
**Growth at pH**				
3	+	+	+	−
4	+	+	+	−
4.5	+	+	+	−
5.5	+	+	+	+
6	+	+	+	+
6.5	+	+	+	+
7	+	+	+	+
7.5	+	+	+	+
8	+	+	+	+
9	+	+	+	−
**Growth in NaCl**				
3% NaCl	+	+	+	−
6.5% NaCl	+	−	−	−
**Carbohydrate fermentation**				
Esculine	+	+	−	−
Cellobiose	+	+	w	+
Maltose	+	+	+	+
Mannitol	w	+	+	−
Salicin	+	+	w	−
Sorbitol	+	+	−	−
Saccharose	+	+	+	+
D-Raffinose	+	+	+	+

*Ho, homo-fermentation; He, hetero-fermentation; w, weak; +, positive; −, negative.*

### Forage Characteristics Before Ensiling

The chemical composition and microbial population by plate culture before ensiling were shown in Table 2. Before ensiling, the DM content of alfalfa was 41.83% and WSC content was 20.37 g/kg DM. The epiphytic population of LAB, yeast, and mold were 3.97, 3.98, and 5.70 log cfu/g FM, respectively.

**TABLE 2:** Chemical composition and microbial population of alfalfa by plate culture before ensiling.

**Items**	**Alfalfa**
DM (%)	41.83
CP (g/kg DM)	237.0
NDF (g/kg DM)	437.8
ADF (g/kg DM)	335.5
WSC (g/kg DM)	20.37
pH	5.84
LAB (log cfu/g FM)	3.97
Yeast (log cfu/g FM)	3.98
Mold (log cfu/g FM)	5.70

*FM, fresh material; DM, dry matter; CP, crude protein; NDF, neutral detergent fiber; ADF, acid detergent fiber; WSC, water-soluble carbohydrates; LAB, lactic acid bacteria; cfu, colony forming units.*

### Effects of Lactic Acid Bacteria on Fermentation Quality, Chemical Composition, and Microbial Population by Plate Culture

The fermentation quality of alfalfa after 30 and 60 days of ensiling was shown in Table 3. The interactions between ensiling days and treatments had a significant impact (*P* < 0.05) on the ratio of LA to AA (LA/AA). The factor of ensiling days significantly influenced (*P* < 0.05) pH, LA/AA, the contents of LA, BA, and NH_3_–N. The factor of treatments had highly significant effect (*P* < 0.01) on pH, LA, and LA/AA. After 30 days of ensiling, the silages inoculated with selected strains had lower (*P* < 0.05) pH than control. Besides, the pH of F1, F50, and L100 treatments were lower (*P* < 0.05) than that of commercial inoculant GFG-treated silage. The contents of LA in F1 and L100 treatments were higher (*P* < 0.05) than that of control and GFG-treated silage. L100-treated silage showed the highest (*P* < 0.05) LA/AA in all treatments. BA was not detected in F1-treated silage. Among all silages, lower (*P* < 0.05) content of NH_3_-N was observed in F1-treated silage. After 60 days of ensiling, all the inoculated silage samples had lower (*P* < 0.05) pH than control, and F1-treated silage showed the lowest pH value (4.60). Compared with control, higher (*P* < 0.05) content of LA in F1, F50, and L100 treatments were observed.

**TABLE 3:** Fermentation quality of alfalfa after 30 and 60 days of ensiling.

**Items**	**Treatments (T)**																
	**30 days (D)**							**60 days (D)**							**ANOVA**		
	**Control**	**GFG**	**F1**	**F50**	**L100**	**L120**	**SEM**	**Control**	**GFG**	**F1**	**F50**	**L100**	**L120**	**SEM**	**D**	**T**	**D × T**
pH	5.41^a^	5.21^ab^	4.70^d^	4.84^cd^	4.83^cd^	5.01^bc^	0.09	5.34^A^	4.81^B^	4.60^D^	4.71^C^	4.70^C^	4.89^B^	0.03	^∗∗^	^∗∗^	NS
LA (g/kg DM)	22.04^d^	25.64^cd^	35.17^ab^	30.40^bc^	38.79^a^	24.53^cd^	2.18	20.60^B^	32.73^AB^	40.78^A^	37.97^A^	43.40^A^	31.32^AB^	4.45	^∗^	^∗∗^	NS
AA (g/kg DM)	29.46^b^	34.12^ab^	37.22^ab^	37.20^ab^	36.28^ab^	38.28^a^	2.36	27.32	28.81	33.99	38.28	31.74	37.16	4.09	NS	NS	NS
LA/AA	0.75^bc^	0.75^bc^	0.95^ab^	0.82^bc^	1.07^a^	0.66^c^	0.06	0.76^E^	1.15^BC^	1.20^B^	0.99^CD^	1.37^A^	0.84^DE^	0.05	^∗∗^	^∗∗^	^∗^
PA (g/kg DM)	ND	ND	ND	ND	ND	0.11	0.04	ND	3.75	0.13	0.14	ND	0.43	1.53	NS	NS	NS
BA (g/kg DM)	0.40^ab^	1.22^a^	ND^b^	0.80^ab^	0.37^ab^	0.35^ab^	0.31	3.45	3.14	1.21	1.12	3.97	1.11	1.52	^∗∗^	NS	NS
NH_3_-N (g/kg TN)	48.15^a^	56.21^a^	24.55^b^	44.08^a^	49.57^a^	47.03^a^	5.91	71.82	86.04	65.27	43.63	53.91	72.32	13.21	^∗∗^	NS	NS

*DM, dry matter; LA, lactic acid; AA, acetic acid; LA/AA, the ratio of lactic acid to acetic acid; PA, propionic acid; BA, butyric acid; TN, total nitrogen; ND, not detected; –, not analyzed; D, ensiling days; T, treatments; D × T, the interaction between ensiling days and treatments; SEM, standard error of the mean; a–e or A–E means values within the same row under same ensiling days with different superscripts in lowercase letter or capital letter differ significantly from each other at *P* < 0.05; ^∗^ and ^∗∗^, significant at *P* < 0.05 and 0.01, respectively; NS, no significant.*

The chemical composition and microbial population by plate culture after 30 and 60 days of ensiling were shown in Table 4. With ensilage time prolonged, the content of CP was also increased (*P* < 0.01). However, the content of WSC and the number of LAB were significantly decreased (*P* < 0.05) by ensiling days. The number of yeast and mold in all silages were below the detectable level (<log 2 cfu/g FM) at both 30 and 60 days. After 30 days of ensiling, the number of LAB ranged around 10^6^–10^8^ cfu/g FM in all silages. The number of LAB was lower (*P* < 0.05) in GFG and F50-treated silages than others at 60 days. There was no significant difference (*P* > 0.05) regarding the contents of DM and NDF.

**TABLE 4:** Chemical composition and microbial population by plate culture of alfalfa after 30 and 60 days of ensiling.

**Items**	**Treatments (T)**																
	**30 days (D)**							**60 days (D)**							**ANOVA**		
	**Control**	**GFG**	**F1**	**F50**	**L100**	**L120**	**SEM**	**Control**	**GFG**	**F1**	**F50**	**L100**	**L120**	**SEM**	**D**	**T**	**D × T**
DM (%)	41.19	41.21	43.62	41.36	42.31	42.78	13.07	41.80	42.61	42.87	40.51	42.42	44.77	8.99	NS	NS	NS
CP (g/kg DM)	229.63^b^	236.61^ab^	246.84^a^	235.65^b^	234.45^b^	237.91^ab^	3.36	266.19^A^	249.48^BC^	247.91^BC^	259.47^AB^	247.17^BC^	242.55^C^	4.38	^∗∗^	NS	^∗∗^
NDF (g/kg DM)	408.05	413.30	412.27	434.21	413.10	433.92	10.23	436.41	412.84	404.88	416.74	422.32	414.20	10.93	NS	NS	NS
ADF (g/kg DM)	315.81^bc^	314.32^bc^	304.12^c^	337.36^ab^	330.23^ab^	339.71^a^	7.00	332.96	325.90	320.11	322.29	327.00	322.21	5.66	NS	NS	^∗^
WSC (g/kg DM)	5.67^b^	6.88^a^	6.81^a^	6.40^ab^	6.15^ab^	6.48^ab^	0.32	5.91	5.45	5.38	6.25	5.89	6.02	0.45	^∗^	NS	NS
LAB (log cfu/g FM)	8.20	8.11	7.43	7.92	7.56	6.50	0.71	6.96^AB^	5.97^C^	6.70^B^	6.03^C^	7.44^A^	6.80^B^	0.19	^∗^	NS	NS
Yeast (log cfu/g FM)	<2.00	<2.00	<2.00	<2.00	<2.00	<2.00	–	<2.00	<2.00	<2.00	<2.00	<2.00	<2.00	–	–	–	–
Mold (log cfu/g FM)	<2.00	<2.00	<2.00	<2.00	<2.00	<2.00	–	<2.00	<2.00	<2.00	<2.00	<2.00	<2.00	–	–	–	–

*FM, fresh matter; DM, dry matter; CP, crude protein; NDF, neutral detergent fiber; ADF, acid detergent fiber; WSC, water-soluble carbohydrates; LAB, lactic acid bacteria; cfu, colony forming units; ND, not detected; –, not analyzed; D, ensiling days; T, treatments; D × T, the interaction between ensiling days and treatments; SEM, standard error of the mean; a–e or A-E means values within the same row under same ensiling days with different superscripts in lowercase letter or capital letter differ significantly from each other at *P* < 0.05; ^∗^and ^∗∗^, significant at *P* < 0.05 and 0.01, respectively; NS, no significant.*

### OTU and Bacterial Community Analysis of Silage

The 16S rDNA fragments covering the variable V3 and V4 regions were PCR amplified and sequenced. As listed in Table 5, the valid sequences per sample ranged from 30,790 to 40,254 were obtained, and these reads were clustered into a total of 1,895 the observed richness OTUs at 97% sequence similarity. The coverage values of all samples were >0.99, suggesting that the sequencing depth was sufficient to reveal the complete bacterial diversity of the samples.

**TABLE 5:** Alpha diversity of bacterial diversity of silages at the 0, 30, and 60 days of ensiling.

**Days**	**Sample**	**Reads**	**OTU**	**Chao**	**Shannon**	**Simpson**	**Coverage**
0	Pre	37,896	114	196.3	1.77	0.31	0.9989
30	Control	31,570	119	146.8	2.65	0.12	0.9989
	GFG	32,321	153	202.5	2.57	0.13	0.9986
	F1	40,046	133	174.0	1.36	0.53	0.9989
	F50	37,351	172	208.4	2.38	0.20	0.9988
	L100	38,760	165	206.0	2.11	0.21	0.9989
	L120	31,019	140	155.8	2.25	0.24	0.9992
60	Control	40,254	127	154.3	2.74	0.11	0.9994
	GFG	33,282	176	185.5	2.24	0.25	0.9994
	F1	38,511	108	137.3	1.75	0.37	0.9993
	F50	33,987	145	201.9	2.32	0.16	0.9986
	L100	38,083	198	231.0	2.64	0.16	0.9991
	L120	30,790	145	169.4	2.62	0.11	0.9988

*Pre, pre-ensiled alfalfa.*

**FIGURE 1:**
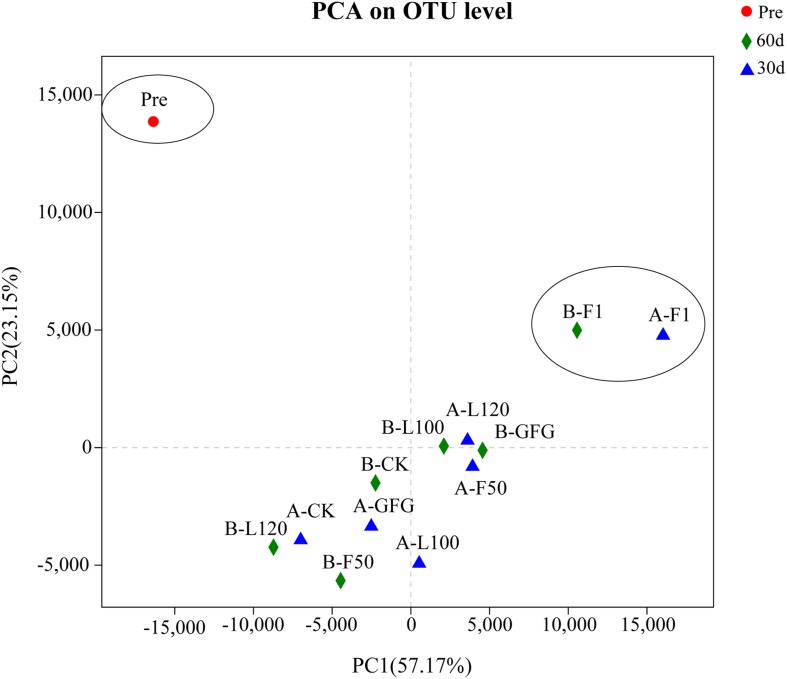
PCA analysis (principle component) based on OTU level in alfalfa silage. PC1, principle component 1; PC2, principle component 2; Pre, pre-ensiled alfalfa; A, 30 days; B, 60 days; CK, control.

The Shannon estimates of diversity indices, the Simpson indices, and the Chao indices ranged from 1.36 to 2.74, 0.11 to 0.53, and 137.3 to 231.0, respectively. At 60 days of ensiling, F1-treated silage showed the lowest OUT and Chao index trend of bacterial richness in all samples. At both sampling days, F1-treated silages had the lowest Shannon index trend of bacterial diversity and the highest Simpson index trend of bacterial evenness. PCA revealed that component 1 and component 2 could explain 57.17 and 23.15% of the total variance, respectively (Figure 1). Three well-separated groups of samples were formed. Only Pre sample was in the first quadrant. A clear distinction between F1 and other inoculated silages at 30 and 60 days was noted, indicating that microbial communities of F1-treated silages could be well separated from other silages.

**FIGURE 2:**
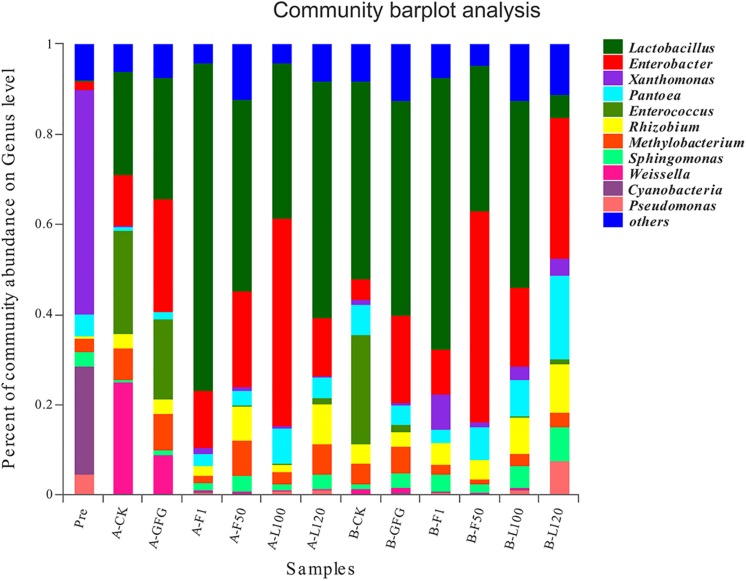
The relative abundance of microbial community at the genus level of pre-ensiled alfalfa and silages after 30 and 60 days of ensiling. Pre, pre-ensiled alfalfa; A, 30 days; B, 60 days; CK, control.

Bacterial community based on genus-level classification was shown in Figure 2. Before ensiling, the dominant genus included *Xanthomonas* (50.02%), *Cyanobacteria* (23.85%), *Pantoea* (4.78%), *Pseudomonas* (4.60%), *Sphingomonas* (3.26%), and *Methylobacterium* (2.93%). However, after ensiling, *Xanthomonas* (0.15–7.67%) and *Cyanobacteria* (0.10–0.20%) both decreased greatly. *Lactobacillus* (5.12–72.74%) and *Enterobacter* (4.62–46.11%) were predominant genera in all silages. Although LAB were detected at a negligible level (<0.01%) in the pre-ensiled alfalfa, the abundance of *Lactobacillus*, *Enterococcus*, and *Weissella* increased greatly after ensiling. *Enterococcus* increased to 23.10–24.90% in the control and 1.63–17.96% in GFG-treated silages, while it increased to 0.09–1.21% in others. *Weissella* was mainly found in the control (24.84%) and GFG-treated silage (8.79%) after 30 days of ensiling, but its abundance reduced to 1.15–1.17% after 60 days of ensiling. The *Lactobacillus* abundance in F1-treated silage increased to 60.32%, while that of others increased to 5.12–47.64% after 60 days of ensiling.

### Relationship Between Microorganism and Parameter Factors

**FIGURE 3:**
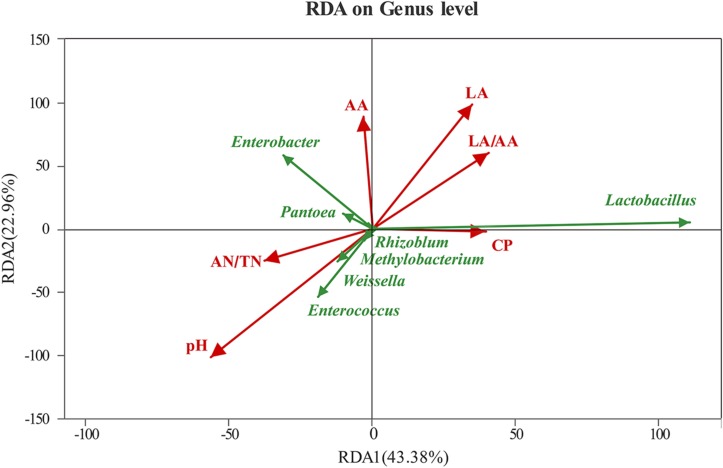
RDA (redundancy analysis) ordination diagram related to the bacterial with explanatory (e.g., pH, AN/TN, LA, AA, LA/AA, CP) variables represented as arrows. RDA1, redundancy analysis 1; RDA2, redundancy analysis 2; AN/TN, NH_3_-N (% total nitrogen); LA, lactic acid; LA/AA, the ratio of lactic acid to acetic acid; CP, crude protein.

The RDA showed that the RDA1 and RDA2 could explain 43.38 and 22.96% of the total variation, respectively (Figure 3). The bacterial community was greatly affected by pH, followed by LA, LA/AA, AA, CP, and AN/TN. The pH and AN/TN were negatively correlated with LA, LA/AA, and CP. Among the top seven genus in abundance, *Lactobacillus* showed positive correlations with LA, LA/AA, and CP and negative correlation with pH and AN/TN. However, *Enterococcus*, *Weissella*, *Methylobacterium*, and *Rhizoblum* showed negative correlations with LA, LA/AA, and CP and positive correlation with pH and AN/TN. The abundance of *Enterobacter* and *Pantoea* had negative correlations with LA/AA and CP.

### Effects on *in vitro* Rumen Fermentation

The effects on IVDMD and GP kinetics were shown in Table 6. Samples inoculated with additives showed higher (*P* < 0.01) value of GP_72_ than control. Compared with control, inoculated with F1 and GFG enhanced (*P* < 0.05) AGPR. F1-treated silage had the highest (*P* < 0.05) IVDMD and asymptotic GP (A) among all treatments.

**TABLE 6:** Gas production and *in vitro* digestibility of silages after 60 days of ensiling.

**Items**	**Control**	**GFG**	**F1**	**F50**	**L100**	**L120**	**SEM**	**ANOVA**
GP_72_ (mL/g)	35.83^d^	38.50^bc^	40.47^a^	37.80^c^	39.90^ab^	39.00^abc^	0.58	^∗∗^
*A* (mL)	32.87^c^	34.00^bc^	35.16^a^	33.48^bc^	34.19^ab^	33.52^bc^	0.35	^∗^
*c* (mL/h)	0.09	0.09	0.09	0.09	0.09	0.09	< 0.01	NS
AGPR (mL/h)	1.02^b^	1.10^a^	1.13^a^	1.08^ab^	1.08^ab^	1.09^ab^	0.02	^∗^
Lag (h)	0.25	0.27	0.36	0.41	0.33	0.40	0.06	NS
IVDMD (g/kg DM)	559.33^b^	566.97^b^	602.67^a^	544.23^b^	531.10^b^	486.53^c^	11.28	^∗∗^

*GP_72_, cumulative gas production of 72 h; *A*, asymptotic gas production; *c*, fractional rate for the gas production of “*A*”; AGPR, average gas production rate; Lag, initial delay time of gas production; IVDMD, *in vitro* dry matter digestibility. ^*a*– *d*^Values within the same row with different superscripts in lowercase letter differ significantly from each other at *P* < 0.05; ^∗^and ^∗∗^, significant at *P* < 0.05 and 0.01, respectively; NS, no significant.*

## Discussion

Dairy cows are regarded as one of the world’s most common domestic animals, as they can provide milk and other resource of dairy products for people. Alfalfa silage has gradually become one of the most important fodders for dairy cows. Hence, it is meaningful to improve the silage quality of alfalfa. In this study, we aimed to select the LAB isolated from feces and rumen fluid of dairy cows and examine their effects on the fermentation quality of alfalfa silage.

The selected strains F1 and F50 showed extensive tolerance to pH, temperature, and salt, in particular F1 expressing better adaption potential to acidity and concentration of salt. L120 was selected by the high rate of growth and acid production ability at 6 h of culture (data was not shown), as the fast accumulation of LA and decline of pH value is crucial in obtaining high-quality silage (Ni et al., 2017b).

Before ensiling, the WSC content of the raw material is an important factor in determining the silage fermentation. The WSC content for assuring acceptable fermentation quality is 60–80 g/kg (Wang et al., 2018). However, low WSC content (20.37 g/kg DM) was detected in our research, so silage could not accumulate LA and reduce pH quickly, resulting in low silage quality (Herrmann et al., 2015). Therefore, it is necessary to inoculate high efficiency fermentation strain to improve silage quality.

The pH of silages inoculated with the selected LAB strains were significantly lower and the LA content were significantly higher than control (except L120-treated silages), which is consistent with the results of Ni et al. (2015), Zheng et al. (2017), and Assar et al. (2018). However, different LAB strains performed different effects on the ensiling process of alfalfa. The LA/AA reflects the relationship between homo-fermentation and hetero-fermentation of LAB (Jones et al., 1992). Our results showed that after 60 days of ensiling, the content of LA was higher than that of AA in F1, L100, and GFG samples, indicating that their fermentation process tended to the direction of homo-fermentation. Among all selected LAB, F1 treatments had the lowest (*P* < 0.05) pH value and the highest (*P* < 0.05) LA content both at 30 and 60 days. The similar result was also detected by Li J.F. et al. (2018) in which LAB from yak increased the LA content and decreased pH compared with control. The content of NH_3_–N in silage is indicative of protein breakdown (Wang et al., 2019). Umana et al. (1991) suggested that well-preserved silages should contain NH_3_–N < 100 g/kg total N. The NH_3_–N content of all silages was below the recommended level. Unexpectedly, compared with 30 days of ensiling, higher CP content were obtained after 60 days of ensiling (*P* < 0.01). This result was agreed with the report of Wang et al. (2018) who found that the CP increased after prolonged ensiling time. But the behind reason needs to be further classified.

Principal component analysis showed that only Pre sample was in the first quadrant, indicating that the microbial communities of fresh materials differed significantly with silages. The bacterial diversity (OTU) of alfalfa increased after ensiling, illuminating that ensiling was the main factor affecting anaerobic fermentation, and the similar result was also reported by Zhao et al. (2017). In addition, close distance of PCA in F1 samples represented the relatively uniform community of bacteria after 30 and 60 days of ensiling.

Before ensiling, *Xanthomonas* and *Cyanobacteria* were two predominant genus in this study. However, their relative abundance approached to a marginal level in all silages after ensiling, indicating that they were inhibited during the ensiling process. *Cyanobacteria* may lead to an overwhelming antioxidant capacity and then accelerate cell death in both animals and plants (Mcelhiney et al., 2001). *Xanthomonas* was also undesirable bacteria for silage fermentation. The roles of *Methylobacterium*, *Pantoea*, *and Pseudomonas* in silage have not been extensively studied. However, they are considered undesirable bacteria which could lead to low silage quality (Mcelhiney et al., 2001; Ni et al., 2017a; Zheng et al., 2017).

The bacterial community was usually dominated by *Lactobacillus* in well-fermented silages (Hao et al., 2018; Xu et al., 2018; Yan et al., 2019). It is well established that *Lactobacillus* plays a critical role in silage fermentation by producing LA, reducing pH, and decreasing the relative abundance of undesirable bacteria. In this study, *Lactobacillus* abundance was the highest in F1-treated silage, which could explain that it had a positive effect on the fermentation quality. *Enterococcus* and *Weissella* belong to cocci-shaped LAB, and they can initiate lactic fermentation in the early stage of ensiling process (Cai et al., 1998). *Enterococcus* mainly appeared in the control silage after 60 days of ensiling, which was much higher than *Lactobacillus* species-inoculated silages. That result might indicate that *Enterococcus* could be outcompeted by *Lactobacillus* at low pH (Ogunade et al., 2018). Although the *Weissella* abundance was relatively high in control and GFG silage at 30 days, it decreased to a marginal level at 60 days. *Weissella* belongs to strictly hetero-fermentative bacteria, producing a mixture of LA and AA by metabolizing WSC (Graf et al., 2016). Previous study has shown that *Enterobacter* decreased rapidly during the ensiling process, and its presence could cause nutrition loss (Pedroso et al., 2010). However, it was shown that *Enterobacter* was one of the two dominant bacteria after ensiling, especially in LAB-treated silage. Although Santos et al. (2016) found that most *Enterobacter* were non-pathogenic when detected in silages, *Enterobacter* could survive under low pH environment and compete with LAB for WSC (Pereira et al., 2007). Similar result has been reported by Li D.X. et al. (2018) in which *Enterobacter* was shown to be the subdominant bacteria after ensiling. RDA analysis suggested that *Lactobacillus* might be the major genus leading to the increase of LA and LA/AA, the decrease of pH and high content of CP conservation. Similarly, Yang et al. (2019) observed that *Lactobacillus* was positively correlated with LA and LA/AA, and negatively correlated with pH and AN/TN. In agreement with our results, Ogunade et al. (2018) found *Methylobacterium* had a positive correlation with pH.

*In vitro* DM disappearance is the result of microbial decomposition of nutrients in silage (Xu et al., 2017). High digestibility is one of good indicators of animal production performance, which is associated with well-fermented silages containing high content of LA (Wilkins et al., 1971; Buxton and Mertens, 1995; Contreras Govea et al., 2016; Li D.X. et al., 2018; Wang et al., 2019). Our study showed that silage inoculated with F1 had the higher IVDMD (*P* < 0.05) than control, which was possibly due to the higher LA preservation. However, the effects on *in vitro* ruminal fermentation varied from LAB additives, only specific LAB inoculant of silage could enhance rumen digestibility (Muck et al., 2007). Compared with GFG, F1 isolated from dairy cows increased the IVDMD, which might be related to their different competitiveness and fermentation products in silage. Another good indirect indicator of fermentation kinetics was regarded as GP. Fermentation gases were produced from a wide range of endogenous and exogenous substrates fermented by microorganisms in the rumen (Zhang et al., 2017). GP is highly dependent on the availability of soluble components. In this study, the GP_72_ of alfalfa silage inoculated with F1 was higher than that with GFG and control. It might be explained by that F1-treated silage had more soluble components and was more conducive to substrate degradation by microorganism in rumen than GFG-treated silage. In the future, we will perform the feeding experiments of the alfalfa silage inoculated with strain F1, and explore its effect on animal performance.

## Conclusion

In this work, 104 LAB strains were isolated from rumen fluid and feces of dairy cows, of which *L. plantarum* F1 and F50, *L. salivarius* L100, and *L. fermentum* L120 were selected to evaluate their effects on silage quality of alfalfa. These four strains could improve the silage quality of alfalfa indicated by lower pH compared with control. The silage treated with F1 isolated from feces showed relatively high *Lactobacillus* abundance and digestible ability than control and the commercial inoculant isolated from forage. Our research further confirmed that LAB strains isolated from animal could be a feasible way for improving the silage quality.

## Data Availability Statement

The datasets generated for this study can be found in Sequence Read Archive (SRA), PRJNA542056, https://www.ncbi.nlm.nih.gov/sra/PRJNA542056.

## Ethics Statement

The animal care and use was conducted in accordance with practices outlined in the Guide for the Care and Use of Agriculture Animals in Agriculture Research and Teaching.

## Author Contributions

LG, DL, DY, and FY designed the study and wrote the manuscript. DY and DL performed the experiments. LG and DY conducted the statistical and bioinformatics analysis. YL, SB, KN, and FY were involved in the revision of the manuscript. All the authors reviewed and approved the final manuscript.

## Conflict of Interest

The authors declare that the research was conducted in the absence of any commercial or financial relationships that could be construed as a potential conflict of interest.

## Footnotes

1 http://drive5.com/uparse/

2 http://www.mothur.org/wiki/Classify.seqs

3 http://rdp.cme.msu.edu/

4 https://www.r-project.org/
